# Hydrothermal CO_2_ Reduction by Glucose as Reducing Agent and Metals and Metal Oxides as Catalysts

**DOI:** 10.3390/molecules27051652

**Published:** 2022-03-02

**Authors:** Maira I. Chinchilla, Fidel A. Mato, Ángel Martín, María D. Bermejo

**Affiliations:** High Pressure Process Group, Department of Chemical Engineering and Environmental Technology, BioEcoUva Research Institute on Bioeconomy, Universidad de Valladolid, 47011 Valladolid, Spain; mairaivette.chinchilla@alumnos.uva.es (M.I.C.); fidel@iq.uva.es (F.A.M.); mamaan@iq.uva.es (Á.M.)

**Keywords:** hydrothermal reaction, CO_2_ conversion, glucose, metal catalysts, metal oxide catalysts

## Abstract

High-temperature water reactions to reduce carbon dioxide were carried out by using an organic reductant and a series of metals and metal oxides as catalysts, as well as activated carbon (C). As CO_2_ source, sodium bicarbonate and ammonium carbamate were used. Glucose was the reductant. Cu, Ni, Pd/C 5%, Ru/C 5%, C, Fe_2_O_3_ and Fe_3_O_4_ were the catalysts tested. The products of CO_2_ reduction were formic acid and other subproducts from sugar hydrolysis such as acetic acid and lactic acid. Reactions with sodium bicarbonate reached higher yields of formic acid in comparison to ammonium carbamate reactions. Higher yields of formic acid (53% and 52%) were obtained by using C and Fe_3_O_4_ as catalysts and sodium bicarbonate as carbon source. Reactions with ammonium carbamate achieved a yield of formic acid up to 25% by using Fe_3_O_4_ as catalyst. The origin of the carbon that forms formic acid was investigated by using NaH^13^CO_3_ as carbon source. Depending on the catalyst, the fraction of formic acid coming from the reduction of the isotope of sodium bicarbonate varied from 32 to 81%. This fraction decreased in the following order: Pd/C 5% > Ru/C 5% > Ni > Cu > C ≈ Fe_2_O_3_ > Fe_3_O_4_.

## 1. Introduction

Global warming is still one of the main worldwide concerns in the present time [1,2]. Increasing the efficiency of processes, implementing renewable sources of energy and fuel switching are some of the alternatives for the reduction of greenhouse emissions [3]. However, in the transition to a decarbonated economy, oil and gas still play a relevant role in energy generation for electricity and transportation; in this context, technological initiatives as carbon capture and utilization become of high interest to mitigate these emissions by using CO_2_ to synthesize high value-added products [4].

CO_2_ is attractive as a raw material for industry because it is cheap, has very low toxicity, is available in great quantity [5] and can be used as feedstock for different processes. Physical methods are widely used to revalorize CO_2_ as refrigerant, solvent, dry ice, etc. Chemical methods are also used to convert it into valuable compounds such as urea and DME [6,7] One of the main difficulties faced in the chemical conversion of CO_2_ is the high thermodynamic stability of the compound [8]. Electrochemical and photochemical reduction for CO_2_ hydrogenation have shown favorable results in overcoming this issue [9]. However, the high costs and low yields of these techniques [10] have led to the study of other alternatives such as the hydrothermal treatment in which CO_2_ reduction takes places in water media at high pressures and temperatures [11,12,13]. In this process, water acts as hydrogen donor instead of H_2_, which is flammable and complex to store [14].

In these processes, CO_2_ is captured in the aqueous media in basic conditions. In most works, it is captured as NaHCO_3_, but it can be also in the shape of carbamates that are formed when CO_2_ is captured by ammonia or amines. This process opens the possibility to connect carbon capture in basic solutions directly with the CO_2_ conversion process, avoiding costly intermediate separation steps [12,15].

In the hydrothermal reduction of CO_2_, the most frequently obtained product is formic acid. This is a compound of great interest for the energy sector because it is an alternative source of hydrogen and can be used directly as an energy-dense carrier for fuel cells [16]; besides, it is biodegradable and less flammable than other fuels at room temperature [17]. 

In order to reduce CO_2_, several metals have been suggested as CO_2_ reductants: Zn [18], Fe [19], Mn [20], Mg [20] and Al [20] (efficiency: Al > Zn > Mn > Fe) [10,21] are mentioned as favorable for the process. Metallic catalysts metals such as Ni-ferrite [22], Ni nanoparticles [23], Ni [24], Raney Ni [25], Cu [20,21], Fe_2_O_3_ [26], Ru/C [27] and Pd/C [12,27] can be used as-is or coupled with “zero-valent” metals to improve the reaction; however, the reduction of the metal after the process in order to recycle the material should be considered.

Organic compounds containing alcohol groups, such as isopropanol [8], glycerol [28], glucose, C2 and C3 alcohols, saccharides and lignin derivatives [29], are often used as reductants as well. It is known that many of these molecules can be obtained from the hydrolysis of lignocellulosic biomass in hydrothermal media.

Subcritical water can act as a basic or acidic catalyst; it has a higher ion product and lower dielectric constant than room-temperature water. In these conditions, the cellulose, hemicellulose and lignin from biomass can be isolated and depolymerized into monomeric units (mainly sugars or phenols). Alongside the decarbonization approach, the usage of biomass is of great interest mainly because of the valorization of lignocellulosic residues that can be converted into several intermediate products such as lactic acid, acetic acid and vanillin [30,31]. Carrying out the hydrolysis of biomass simultaneously with the reduction of CO_2_ captured as a basic solution (i.e., bicarbonate, amine carbamates of ammonia) could be interesting because many of the hydrolysis products of biomass contain alcohol groups that can act as reductants of CO_2_ in hydrothermal media.

So far, there has been a number of studies in which the use of catalysts (Cu, Ni, Pd/C) in hydrothermal CO_2_ reduction was carried out using metals as reductants (Zn, Mg, Al, etc.) [12,18,20,21,22,25]. This work studies, for the first time, the influence of different catalysts in the hydrothermal reduction of CO_2_ by using an organic (glucose) as a reducing agent. As CO_2_ source, sodium bicarbonate (NaHCO_3_) and ammonium carbamate (NH₄[H₂NCO₂]) were used. There are literature studies stating that ammonium carbamate is reduced by using metals or hydrogen [12], but this is the first time that the reduction is performed using organics containing an alcohol group. The main objective of the present work is to develop batch screening reactions to find the best catalyst that can improve the formic acid production, as well as lowering the temperature for the reduction reactions normally fixed at 300 °C in other works [29]. In addition, as formic acid can be also derived by sugar hydrolysis [32,33,34,35], experiments to study the origin of the carbon forming formic acid by using NaH^13^CO_3_ as carbon source were performed.

## 2. Results

To perform the reduction of CO_2_, several experiments were carried out by using glucose as organic reductant, sodium bicarbonate (SB) and ammonium carbamate (AC) as sources of carbon and several metals and metal oxides as catalysts (Cu, Ni, Pd/C 5%, Ru/C 5%, Fe_2_O_3_ and Fe_3_O_4_). Activated carbon (C) was used in some experiments in order to compare its performance with the palladium and ruthenium supported catalysts.

It should be noted that only the products of the liquid phase were analyzed. Gas products were not formed or were produced in so small amounts that they could not be collected. It is not excluded that gases such as CH_4_ [23,24] could be produced in a very small amount in the case of Ni catalyst.

### 2.1. Particle Size of the Catalysts

**Figure 1:**
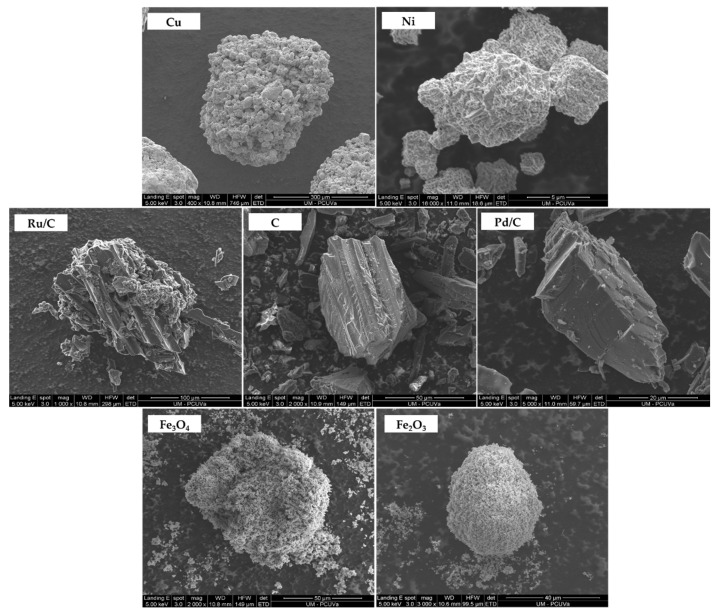
SEM images of the catalyst particles.

As seen in Figure 1, SEM images of the unreacted catalysts were taken in order to measure the average particle size. The approximate diameters of the particles of each the catalysts before the hydrothermal reaction were as follows: Cu: 400 µm; Ni: 9 µm; Pd/C: 25 µm; C: 55 µm; Ru/C: 175 µm; Fe_2_O_3_; Fe_3_O_4_: 93 µm.

### 2.2. Results of Hydrothermal Reactions with Sodium Bicarbonate as Carbon Source

In the reaction of SB with glucose, typical products derived from the hydrothermal reduction of glucose were observed [29,33,34,35,36,37]. Formic acid (FA), acetic acid (AA) and lactic acid (LA) were the main compounds produced in the reactions. In minor amounts, glyceraldehyde, glycolaldehyde, formaldehyde, ethylene glycol, acetone, pyruvaldehyde, galacturonic acid and 5-HMF were obtained. The yields of the three main products of the catalyzed reactions are shown in Table 1, Table 2 and Table 3. Each experiment was repeated at least twice, the average error being around 5%.

After carrying out the reduction of CO_2_ captured as SB, it was found that the highest yields of FA were obtained by using C and Fe_3_O_4_ as catalysts, reaching yields of 53% and 52%, respectively (Table 1). The conditions at which the maximum values were obtained were 200 °C and 30 min of reaction for C and 250 °C and 30 min of reaction for Fe_3_O_4_.

The highest yield for AA was obtained in the sample without catalyst: 45% at 250 °C and 30 min. This was followed by Ni and Cu catalysts, which achieved yields of 45% and 44%, at 250 °C and 30 min and 250 °C and 120 min (Table 2).

For LA, the maximum yield was achieved with Fe_3_O_4_, 43% at 250 °C and 30 min of reaction (Table 3).

It is remarkable that most of the catalysts promoted similar or less yield of FA in comparison to the sample without catalyst; in fact, only C and Fe_3_O_4_ improved the yield of FA over AA and LA over the sample with no catalyst. 

#### Results of Hydrothermal Reactions with Ammonium Carbamate as Carbon Source

As in the previous case, the main products of the reaction with AC were FA, AA and LA. The yields achieved for each compound are shown in Table 4, Table 5 and Table 6. The experiments were repeated at least twice, the average error being around 3%.

After the hydrothermal reduction of AC, it was found that the highest yield of FA was 26% and was obtained by using Fe_3_O_4_ at 200 °C and 120 min (Table 4).

The maximum value for AA was obtained with Ni, 15% at 250 °C and 180 min. This was followed by that obtained with Cu, 14% at 200 °C and 60 min (Table 5).

For LA, the highest value was 16% and was obtained by using Fe_3_O_4_ at 200 °C and 90 min. This was followed by that obtained with C, 13% at 200 °C and 30 min (Table 6).

Once again, Fe_3_O_4_ promoted the maximum yields of FA over AA and LA in comparison to the rest of the catalysts. Only Fe_3_O_4_ and C improved the yield of FA compared to the sample with no catalyst.

In general, the yields of FA, AA and LA obtained by the reduction of AC are much lower (less than 25%) than those observed with SB (less than 53%). Some other works have shown that sodium bicarbonates and carbonates required high-temperature reactions to achieve higher yields of FA. SB and AC are decomposed easily into HCO^3−^, which is the species that is going to be reduced in the reaction. In the case of AC, not only HCO^3−^ is formed. There is another step in which AC is also decomposed because the H^+^ protons of the ion NH_4_^+^ are being donated to other compounds, and then the yield to FA is reduced because there is a competition between two reactions: the reduction of AC and the thermal decomposition of AC [13,16]. It was observed that the experiments held at 200 °C showed higher yields of FA than the reactions at 250 °C. The reduction of CO_2_ is favored by the reaction in alkaline media; when the temperature rises, NH_4_^+^ dissociates into NH_3_ and H^+^, which are species that reduce the alkalinity and might reduce the solubility of CO_2_ in water [10,38].

### 2.3. Nuclear Magnetic Resonance Spectroscopy Results

It is known that FA can be generated from sugars at lower temperatures in basic aqueous media [32,33,34] and can be also obtained by the reduction of SB at temperatures higher than 300 °C [29]. In order to understand the reactions, it is necessary to check whether the FA is coming from SB or from glucose and if the catalysts are favoring or disfavoring one or the other reaction. To do so, experiments with an isotope of sodium bicarbonate (NaH^13^CO_3_; SB-^13^C) were performed with the different catalysts. ^13^C-NMR analyses were carried out to identify the fraction of formic acid that possesses ^13^C, which comes from the reduction of the carbon source, and the fraction that comes from glucose. The experiments were conducted at 250 °C and 2 h.

**Figure 2:**
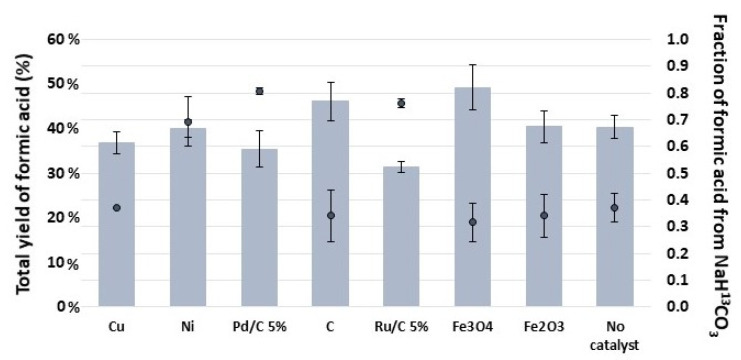
Fractions of formic acid coming from the reduction NaH^13^CO_3_ and from the oxidation of glucose for each of the catalysts at 250 °C and 2 h. Gray bars represent the total yield of formic acid of each sample (obtained by HPLC); black dots represent the fraction of formic acid coming from NaH^13^CO_3_ (obtained by ^13^C-NMR). The average error in the measure of the fraction of formic acid was 5%.

The fraction of formic acid coming from the SB-^13^C when using each of the catalysts is presented in Figure 2.

It was observed that although Fe_3_O_4_ is the catalyst that provides the highest yield of total FA (49%, 250 °C and 2h, measured by HPLC), its proportion of reduced SB-^13^C is lower (0.32) in comparison to the fraction of FA obtained with Pd/C 5%, Ru/C 5% and Ni (0.81, 0.76 and 0.69, respectively).

The metal supported catalysts (Pd/C 5% and Ru/C 5%) presented the highest selectivity in reducing CO_2_ in comparison to the performance of the activated carbon support (C), which reached a fraction of 0.34.

There were catalysts that did not improve the reduction of SB-^13^C; in fact, the reaction without catalyst (fraction FA-^13^C: 0.37) showed a slightly higher capability to reduce CO_2_ than Cu, Fe_3_O_4_ and Fe_2_O_3_ (0.37, 0.32 and 0.34, respectively).

The order in which catalysts were able to reduce CO_2_ captured as SB-^13^C was as follows: Pd/C 5% > Ru/C 5% > Ni > Cu > C ≈ Fe_2_O_3_ > Fe_3_O_4_.

In all the experiments, the only products that came from the direct reduction of carbon source (SB-^13^C) were FA-^13^C at δ = 163 ppm and an unidentified compound at δ = 173 ppm (this peak was absent in Fe_2_O_3_ and in the sample with no catalyst). At δ = 127 ppm, another peak was observed; according to the literature [39,40], this compound could be ^13^CO_2_ dissolved in the sample.

An AA-^13^C standard was injected. AA-^13^C standard peak was observed at δ = 184 ppm, and then the possibility that the unidentified peak at δ = 173 ppm was AA-^13^C was excluded.

#### Possible Mechanisms of Reaction

In the NMR spectra, it was confirmed that the reduction of the carbon source led mostly to the formation of FA, while byproducts and FA were obtained from the oxidation of glucose.

In literature were found some of the possible mechanisms of reaction of glucose at high water temperatures, subcritical and supercritical water [41,42,43,44]. Glucose can be transformed in two different ways: by following a retro-aldol condensation reaction to produce glycolaldehyde or through the isomerization of the glucose into fructose (favored by basic media) which can be dehydrated to form 5-HMF (favored by the acid media) or can produce glyceraldehyde by means of another retro-aldol condensation reaction. Finally, the glyceraldehyde can be isomerized into pyruvaldehyde, which could be a precursor of lactic acid.

Besides the retro-aldol reactions that can lead to the production of lactic acid and glycolaldehyde, 5-HMF can be transformed into formaldehyde and furfural in acid media [43], but in our case, reactions were performed in basic media, so this step may or may not be occurring.

**Figure 3:**
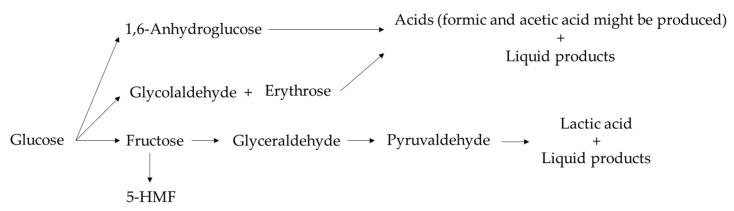
Mechanisms of oxidation of glucose.

Some other works [42] described that glucose can also dehydrate to form 1,6-anhydroglucose. This molecule can be a precursor of acids or can be transformed into D-fructose and follow a reverse aldol condensation reaction to form erythrose and glycolaldehyde that can produce acids as well. In Figure 3, the main mechanisms of oxidation of glucose are represented. According to Kabyemela et al. [42], some of the products derived from the oxidation of glucose that can be identified according to these mechanisms are fructose, erythrose, glyceraldehyde, glycolaldehyde, pyruvaldehyde, dihydroxyacetone, 1,6-anhydroglucose, 5-HMF, acetic acid and formic acid. Kabyemela et al. [42] also have identified some products of the decomposition of fructose such as pyruvaldehyde, erythrose, glyceraldehyde, dihydroxyacetone, acetic acid and formic acid. Erythrose and 1,6-anhydroglucose can also be the precursors of acetic and formic acids.

Glucose has five -OH (hydroxyl) groups. In a previous work, it has been proposed that alcohol groups act as reducing agents for CO_2_ [29]. According to Shen et al., the reduction of the carbon source is mainly due to the alcohol moiety [8,45]. According to other studies, compounds with primary alcohol groups presented slightly higher yields in comparison with compounds with secondary alcohol groups. Because of the steric effects, the position of the hydroxyl group in the compound could be of importance in the reduction of the carbon source [29]. Shen et al. proposed a mechanism of reduction of CO_2_ through alcohol molecules in which, through a cyclic transition state, a H^−^ from the α-carbon of −OH moiety is transferred to the ion bicarbonate and the resulting species dehydrate quickly into formate [8,29,45]. Most of the products of glucose decomposition contain alcohol groups (fructose, glyceraldehyde, glycolaldehyde, lactic acid), and Andérez et al. [29] proved that formic acid is rendered in appreciable yields.

Regarding catalysts, it can be found in the literature that in reactions that use metals as reductants, HCO_3_^-^ is adsorbed on a Pd/C surface, promoting the formation of C_1_ intermediates species to produce FA and traces of CH_4_ and improving the generation of C-C bonds to form C_2_ compounds [46]. Cu and Ni have showed also good performance in reducing HCO_3_^-^ into C_1_ compounds when using metals such as Fe as reducing agents [47,48]. In other studies, experiments with a mix of Fe and Fe_3_O_4_ were performed to reduce CO_2_. In these works, Fe is reduced into Fe_3_O_4_, generating hydrogen. Fe_3_O_4_ is transformed into Fe_3_O_4-x_, and then hydrogen and C=O of HCO_3_^−^ are adsorbed in the surface of the metal oxide and react to produce formic acid [49].

As seen before, catalysts can influence the performance of the hydrothermal reactions for CO_2_ reduction and glucose oxidation.

## 3. Materials and Methods

### 3.1. Chemicals

Ammonium carbamate (AC) (99%), sodium bicarbonate (SB) (100%), sodium bicarbonate ^13^C (SB-^13^C) (100%) and acetic acid ^13^C (AA-^13^C) were used as sources of captured CO_2_. D-(+)-Glucose (100%) was used as reducing agent. Fine powder of commercial Cu, Ni, Pd/C (5 wt% of metal loading), activated carbon, Ru/C (5 wt% of metal loading, 50% water wet paste), Fe_2_O_3_ and Fe_3_O_4_ were used as catalysts. Sodium bicarbonate was purchased from COFARCAS (Spain), Ru/c 5% was provided by Strem Chemicals and the rest of the chemicals were acquired from Sigma-Aldrich. Deionized water was used to prepare the dilutions.

### 3.2. Catalytic Experiments

Two solutions were prepared: the first one contained 0.05 M of glucose and 0.5 M of sodium bicarbonate, and the second one consisted of 0.05 M glucose and 0.5 M ammonium carbamate (ratio 1:10, glucose:carbon source in mol). Hydrothermal reactions for the conversion of CO_2_ (captured as ammonium carbamate and sodium bicarbonate) were carried out in SS316 stainless steel horizontal tubular reactors of 10 mL (internal volume). The batch reactors were filled with liquid up to 45% of the total volume.

Different sets of reactions were performed at temperatures of 200 and 250 °C for 30, 60, 90, 120 and 180 min. The pressure inside the reactor corresponds to the saturation pressure of the set temperature. A fluidized bed heater was used to reach the temperature of the reactions. A temperature probe was installed inside the vessels. Reactors were placed in the heaters, achieving the working temperature within 7 min. Once the residence time was completed, each reactor was immediately extracted and placed in a bath of cold water. All samples were filtered with 0.22 µm Nylon filter and then collected in glass vials to be analyzed by HPLC or NMR spectroscopy.

The yields to formic acid, lactic acid and acetic acid were calculated as follows:

(1) $$Y_{product}=\frac{C_{Product,f}}{C_{Glucose,i}}$$

where *C_Product,f_* is the molar concentration of formic acid at the end of the reaction and *C_Glucose,i_* is the initial molar concentration of glucose.

All the experiments were repeated at least 2 times, and the error was calculated with the standard deviations of the yields.

The amounts of catalyst used were as follows: For Cu and Ni, a molar relation of metal:carbon source of 5:1 was used. For Pd/C 5%, C and Ru/C 5%, 55 wt% catalyst with respect to the initial weight of carbon source was added. For Fe_3_O_4_ and Fe_2_O_3_, a molar relation of metal:carbon source of 1:1 was utilized.

### 3.3. Scanning Electronic Microscopy (SEM)

SEM images of the catalyst were taken in order to corroborate the particle size. An FEI QUANTA 200 FEG (ESEM) was used at high-vacuum operation (<6 × 10^−4^ Pa (4.5 × 10^−2^ Torr)), electron landing energy of 5 keV and spot of 3.0.

### 3.4. High-Pressure Liquid Chromatography (HPLC)

The liquid samples were analyzed by HPLC (Waters, Alliance separation module e2695) with RI detector (Waters, 2414 module) and a Rezex ROA-Organic Acid H+ (8%) column. As mobile phase, 25 mM H_2_SO_4_ was used at 0.5 mL/min of flow rate. The temperatures of the column and the detector were 40 and 30 °C.

### 3.5. Nuclear Magnetic Resonance Spectroscopy (NMR Spectroscopy)

Spectra of all the samples in which SB-^13^C was used as carbon source were recorded on a 500 MHz Agilent instrument equipped with a OneNMR probe. The acquisition parameters for ^13^C NMR spectra were as follows: 25 °C, 70 s relaxation delay between transients, 45° pulse width, spectral width of 31250 Hz, a total of 16 transients and 1.048 s acquisition time. The inverse gated decoupling technique to suppress the nuclear Overhauser effect (NOE) was used to obtain quantitative measurement. The acquisition parameters for ^1^H NMR spectra were as follows: 25 °C, 70 s relaxation delay between transients, 90° pulse width, spectral width of 8012.8 Hz, a total of 4 transients and 2.044 s acquisition time. The sequence PRESAT was used in order to suppress the strong signal of water.

^1^H and ^13^C NMR chemical shifts (δ) were reported in parts per million (ppm) and referenced to tetramethylsilane (TMS).

## 4. Conclusions

In this work, the hydrothermal conversion of CO_2_ captured as sodium bicarbonate and ammonium carbamate was studied. Glucose was used as a reducing agent, and metal and metal oxides (Cu, Ni, Pd/C 5%, Ru/C 5%, Fe_2_O_3_ and Fe_3_O_4_), as well as activated carbon (C), were used as catalysts. The main products of the reaction with ammonium carbamate were formic acid, acetic acid and lactic acid.

The yields of formic acid, acetic acid and lactic acid obtained by the reduction of ammonium carbamate were much lower (less than 25%) than those observed when sodium bicarbonate was used as the carbon source (less than 53%).

For ammonium carbamate experiments, C and Fe_3_O_4_ promoted higher yields of FA over AA and LA in comparison to the rest of the catalysts and improved the yield of FA in comparison to the sample without catalyst.

In the experiments with sodium bicarbonate, C and Fe_3_O_4_ appeared to be the most promising catalysts for improving the yield of formic acid. The origin of the carbon forming formic acid was investigated by using NaH^13^CO_3_. It was found that although C and Fe_3_O_4_ achieved the highest total formic acid yield, they seem to favor the oxidation of glucose instead of the reduction of CO_2_. However, it should be noted that even though Pd/C 5%, Ni and Ru/C 5% yields of total formic acid were lower, they were shown to be more selective in producing formic acid from CO_2_ than the other catalysts. This aspect is important when considering the selection of a catalyst for making a process that primarily promotes a higher conversion of the carbon source.

## Figures and Tables

**Table 1:** Yields of formic acid obtained after the hydrothermal reaction of NaHCO_3_ with glucose in the presence of catalysts. The higher yields obtained after the hydrothermal reactions are marked with an asterisk (*).

**Reaction Temperature: 200 °C**								
**Reaction Time (min)**	**Cu**	**Ni**	**Pd/C**	**C**	**Ru/C**	**Fe_3_O_4_**	**Fe_2_O_3_**	**No Catalyst**
30	44%	44%	20%	* 53%	31%	49%	45%	44%
60	43%	40%	16%	46%	30%	51%	49%	48%
90	43%	35%	18%	51%	25%	48%	45%	-
120	39%	34%	20%	51%	27%	49%	48%	51%
180	39%	32%	20%	37%	23%	46%	41%	40%
**Reaction Temperature: 250 °C**								
**Reaction Time (min)**	**Cu**	**Ni**	**Pd/C**	**C**	**Ru/C**	**Fe_3_O_4_**	**Fe_2_O_3_**	**No Catalyst**
30	41%	41%	29%	50%	38%	* 52%	40%	47%
60	36%	-	31%	47%	33%	44%	42%	41%
90	37%	35%	36%	47%	31%	44%	39%	38%
120	37%	40%	35%	46%	32%	49%	40%	40%
180	32%	34%	38%	46%	26%	45%	40%	39%

**Table 2:** Yields of acetic acid obtained after the hydrothermal reaction of NaHCO_3_ with glucose in the presence of catalysts. The higher yields obtained after the hydrothermal reactions are marked with an asterisk (*).

**Reaction Temperature: 200 °C**								
**Reaction Time (min)**	**Cu**	**Ni**	**Pd/C**	**C**	**Ru/C**	**Fe_3_O_4_**	**Fe_2_O_3_**	**No Catalyst**
30	39%	40%	25%	33%	31%	31%	38%	39%
60	38%	41%	26%	33%	33%	33%	39%	40%
90	40%	39%	23%	34%	35%	35%	39%	40%
120	38%	37%	27%	33%	31%	31%	37%	37%
180	36%	36%	27%	28%	31%	31%	37%	37%
**Reaction Temperature: 250 °C**								
**Reaction Time (min)**	**Cu**	**Ni**	**Pd/C**	**C**	**Ru/C**	**Fe_3_O_4_**	**Fe_2_O_3_**	**No Catalyst**
30	40%	* 45%	26%	33%	34%	34%	41%	* 45%
60	-	-	23%	30%	27%	27%	-	38%
90	43%	42%	24%	35%	30%	30%	39%	38%
120	* 44%	43%	24%	35%	30%	30%	39%	-
180	37%	39%	24%	34%	30%	30%	39%	40%

**Table 3:** Yields of lactic acid obtained after the hydrothermal reaction of NaHCO_3_ with glucose in the presence of catalysts. The higher yields obtained after the hydrothermal reactions are marked with an asterisk (*).

**Reaction Temperature: 200 °C**								
**Reaction Time (min)**	**Cu**	**Ni**	**Pd/C**	**C**	**Ru/C**	**Fe_3_O_4_**	**Fe_2_O_3_**	**No Catalyst**
30	34%	34%	23%	28%	33%	35%	35%	35%
60	31%	31%	22%	26%	32%	34%	34%	34%
90	36%	31%	27%	29%	30%	37%	37%	36%
120	32%	32%	27%	-	29%	34%	34%	34%
180	31%	33%	28%	34%	28%	33%	31%	31%
**Reaction Temperature: 250 °C**								
**Reaction Time (min)**	**Cu**	**Ni**	**Pd/C**	**C**	**Ru/C**	**Fe_3_O_4_**	**Fe_2_O_3_**	**No Catalyst**
30	35%	38%	36%	39%	39%	* 43%	37%	38%
60	33%	-	33%	40%	32%	40%	40%	35%
90	36%	38%	39%	39%	34%	35%	40%	35%
120	34%	39%	34%	38%	34%	38%	37%	38%
180	31%	37%	31%	36%	29%	36%	34%	37%

**Table 4:** Yields of formic acid obtained after the hydrothermal reaction of NH₄[H₂NCO₂] with glucose in the presence of catalysts. The higher yields obtained after the hydrothermal reactions are marked with an asterisk (*).

**Reaction Temperature: 200 °C**								
**Reaction Time (min)**	**Cu**	**Ni**	**Pd/C**	**C**	**Ru/C**	**Fe_3_O_4_**	**Fe_2_O_3_**	**No Catalyst**
30	16%	14%	9%	21%	9%	21%	18%	17%
60	17%	19%	7%	-	-	20%	17%	17%
90	17%	14%	3%	21%	3%	24%	18%	17%
120	14%	7%	3%	16%	3%	* 26%	19%	19%
180	16%	15%	2%	20%	3%	25%	19%	19%
**Reaction Temperature: 250 °C**								
**Reaction Time (min)**	**Cu**	**Ni**	**Pd/C**	**C**	**Ru/C**	**Fe_3_O_4_**	**Fe_2_O_3_**	**No Catalyst**
30	10%	8%	2%	21%	2%	25%	20%	17%
60	9%	3%	3%	15%	1%	24%	17%	16%
90	8%	4%	3%	9%	1%	24%	16%	16%
120	5%	3%	3%	16%	1%	20%	12%	14%
180	5%	1%	3%	15%	1%	9%	12%	14%

**Table 5:** Yields of acetic acid obtained after the hydrothermal reaction of NH₄[H₂NCO₂] with glucose in the presence of catalysts. The higher yields obtained after the hydrothermal reactions are marked with an asterisk (*).

**Reaction Temperature: 200 °C**								
**Reaction Time (min)**	**Cu**	**Ni**	**Pd/C**	**C**	**Ru/C**	**Fe_3_O_4_**	**Fe_2_O_3_**	**No Catalyst**
30	9%	9%	6%	7%	9%	11%	11%	11%
60	* 14%	12%	8%	8%	9%	11%	11%	11%
90	11%	12%	8%	11%	8%	11%	11%	12%
120	11%	10%	9%	9%	9%	12%	11%	12%
180	11%	12%	9%	11%	11%	12%	12%	13%
**Reaction Temperature: 250 °C**								
**Reaction Time (min)**	**Cu**	**Ni**	**Pd/C**	**C**	**Ru/C**	**Fe_3_O_4_**	**Fe_2_O_3_**	**No Catalyst**
30	9%	12%	8%	8%	10%	11%	10%	10%
60	9%	11%	8%	9%	10%	10%	8%	10%
90	10%	13%	9%	6%	11%	11%	11%	11%
120	10%	13%	9%	10%	11%	10%	10%	11%
180	10%	* 15%	10%	11%	13%	11%	10%	11%

**Table 6:** Yields of lactic acid obtained after the hydrothermal reaction of NH₄[H₂NCO₂] with glucose in the presence of catalysts. The higher yields obtained after the hydrothermal reactions are marked with an asterisk (*).

**Reaction Temperature: 200 °C**								
**Reaction Time (min)**	**Cu**	**Ni**	**Pd/C**	**C**	**Ru/C**	**Fe_3_O_4_**	**Fe_2_O_3_**	**No Catalyst**
30	8%	9%	5%	* 13%	5%	10%	9%	9%
60	7%	9%	6%	5%	5%	10%	8%	9%
90	10%	8%	4%	11%	3%	* 16%	10%	11%
120	12%	9%	6%	9%	4%	6%	9%	8%
180	9%	9%	6%	7%	5%	5%	10%	10%
**Reaction Temperature: 250 °C**								
**Reaction Time (min)**	**Cu**	**Ni**	**Pd/C**	**C**	**Ru/C**	**Fe_3_O_4_**	**Fe_2_O_3_**	**No Catalyst**
30	5%	7%	5%	11%	4%	13%	12%	9%
60	8%	4%	4%	11%	4%	6%	9%	10%
90	7%	4%	4%	5%	3%	4%	9%	8%
120	8%	6%	4%	9%	5%	10%	8%	9%
180	6%	5%	4%	10%	3%	10%	8%	10%
